# Arterial versus venous lactate: a measure of sepsis in children

**DOI:** 10.1007/s00431-017-2925-9

**Published:** 2017-06-10

**Authors:** Sahan Asela Samaraweera, Berwyck Gibbons, Anami Gour, Philip Sedgwick

**Affiliations:** 1 Medical School, St. George’s University of London, London, UK; 2 Paediatric Intensive Care Unit (PICU), St. George’s University Hospitals NHS Foundation Trust, London, SW17 0QT UK; 3 Paediatric Intensive Care Unit (PICU), St. George’s University of London, London, SW17 0QT UK; 4 Institute for Medical and Biomedical Education, St. George’s University of London, London, SW17 0QT UK

**Keywords:** Paediatric sepsis, Venous blood lactate, Arterial blood lactate, Blood gas

## Abstract

This study assessed the agreement between arterial and venous blood lactate and pH levels in children with sepsis. This retrospective, three-year study involved 60 PICU patients, with data collected from electronic or paper patient records. The inclusion criteria comprised of children (≤17 years old) with sepsis and those who had a venous blood gas taken first with an arterial blood gas taken after within one hour. The lactate and pH values measured through each method were analysed. There is close agreement between venous and arterial lactate up to 2 mmol/L. As this value increases, this agreement becomes poor. The limits of agreement (LOA) are too large (±1.90 mmol/L) to allow venous and arterial lactate to be used interchangeably. The mean difference and LOA between both methods would be much smaller if derived using lactate values under 2.0 mmol/L. There is close agreement between arterial and venous pH (MD = −0.056, LOA ± 0.121). However, due to extreme variations in pH readings during sepsis, pH alone is an inadequate marker.

*Conclusion*: A venous lactate ≤2 mmol/L can be used as a surrogate for arterial lactate during early management of sepsis in children. However, if the value exceeds 2 mmol/L, an arterial sample must confirm the venous result.

**Table:** 

**What is known:** *• In children with septic shock, a blood gas is an important test to show the presence of acidosis and high lactic acid. Hyperlactataemia on admission is an early predictor of outcome and is associated with a greater mortality risk.* *• An arterial sample is the standard for lactate measurement, however getting a sample may be challenging in the emergency department or a general paediatric ward. Venous samples are quicker and easier to obtain. Adult studies generally advise caution in replacing venous lactate values for the arterial standard, whilst paediatric studies are limited in this area.*
**What is new:** *• This is the first study assessing the agreement between arterial and peripheral venous lactate in children with sepsis, with a significant sample of patients.* *• This study shows that a venous sample with a lactate of ≤ 2 mmol/L can be used as a surrogate measurement for arterial lactate during early management of sepsis in children. However, if the venous lactate is above 2 mmol/L, an arterial sample must be taken to confirm the result.*

## Background

Measurement of lactate has long been considered vital in the assessment of critically ill patients, both as an indicator of severity of illness and as a predictor of mortality [5, 8, 11, 16].

Lactate accumulates due to anaerobic metabolism and reflects the degree of tissue hypoxia due to poor perfusion [8, 9]. Hyperlactataemia occurs in trauma, hypoxaemia, severe anaemia and septic shock, common conditions in Paediatric Intensive Care [1, 2].

In critically ill children, hyperlactataemia on admission is an early predictor of outcome in children with sepsis and is associated with greater mortality risk [8, 15]. Measurement identifies children at higher risk for severe outcomes, but can also monitor improvement and recovery with timely treatment and intervention [6, 9, 15, 16]. A raised lactate must exceed 2 mmol/L to be considered abnormal.

An arterial sample is the standard method of lactate determination [11], and historically, studies have used arterial lactate as the foundation of their hypotheses on sepsis markers and prognostic indicators. In paediatric intensive care, however, venous and capillary blood gases are being increasingly used: arterial blood gases (ABGs) are painful, more invasive and can be difficult to perform, sometimes requiring multiple attempts, which can be distressing to the child [2, 4, 11].

Advocating the use of venous lactate as a surrogate for arterial lactate relies on the proven efficacy of venous blood lactate as a true marker for sepsis. Results from previous adult studies have shown varying agreement or correlation between venous and arterial lactate and pH. Bloom et al. [4] found that ‘the agreement between abnormal PVL (peripheral venous lactate) and AL (arterial lactate) is poor and the high rate of misclassification may suggest that PVL is not a good substitute for AL if the venous lactate is abnormal’. Conversely, Contenti et al. [5] found a strong correlation between arterial and venous blood lactate levels, and concluded that ‘Initial VBL (venous blood lactate) may be used efficiently to assess the severity of sepsis, and it could even be more effective than ABL (arterial blood lactate) to detect the presence of severe sepsis’. Other studies also found a strong correlation or agreement between venous and arterial lactate, but overall advise caution in substituting venous lactate for the arterial gold standard [7, 10, 11, 13, 16].

**Fig. 1:**
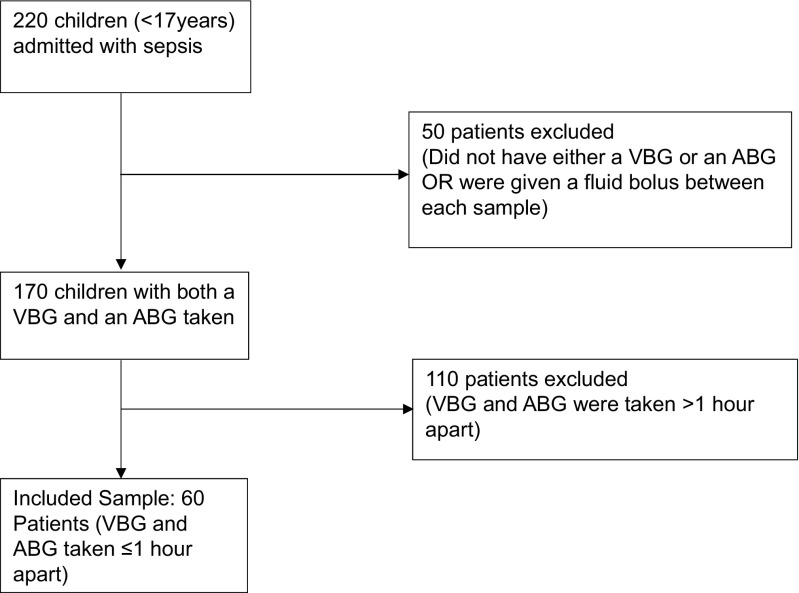
Flowchart of the inclusion process for venous and arterial lactate and pH samples

Studies into the efficacy of venous over arterial lactate in the paediatric population are more limited in number. Murdoch et al. in a small study of seven children found that differences ‘between arterial and mixed venous samples were clinically insignificant’ [12]. In a more comprehensive Paediatric Intensive Care Unit (PICU) study, with children being admitted for multiple causes, Bilan et al. concluded that a venous blood gas (VBG) can be used instead of an ABG in some diseases including sepsis, but in others, particularly cardiac conditions, an ABG is preferable and must not be replaced by a VBG [2]. In addition, Sarmiento et al. compared arterial lactate with central venous lactate in 42 children with sepsis, with or without shock, to find a strong positive correlation between the two measures [14]. Nonetheless, establishing that correlation exists between venous and arterial measurements does not advocate agreement between the measurements. Such an approach is inappropriate and misleading [3].

Interestingly, in studies that have found agreement, patient lactate levels were commonly within normal range under 2 mmol/L. It has been observed that in patients with lactate values over 2 mmol/L, agreement between venous and arterial lactate is poor, leading to the hypothesis that venous lactate should not be a substitute for arterial lactate if the venous value is abnormal [4].

Understanding the level of agreement between arterial and venous lactate, and how this agreement changes as these values increase, will demonstrate whether VBGs could act as a surrogate for ABGs; or alternatively, an ABG should always be sought to confirm a venous measurement when identifying sepsis in children.

## Methods

This was a retrospective, cohort study of paediatric intensive care patients over the course of three years and four months between June 2012 and October 2015. The setting was the PICU department of a large London-based hospital, with an annual intake of approximately 650 patients a year. Data was collected from the trust’s online electronic patient database (‘iCLIP’) or from patient paper notes. As this was a service evaluation, with no study interventions or deviations from usual practice, neither consent nor ethical approval was required.

The inclusion criteria encompassed children with sepsis, neutropenic sepsis and septic shock (≤17 years of age), who had a VBG taken first, then an ABG taken after within one hour. The lactate and pH values measured by each method were analysed. Premature infants, patients admitted without sepsis and those who received a fluid bolus between the two blood gas samples were excluded from this study.

The main objective was to assess the level of agreement between arterial and venous lactate and pH measurements through calculating the mean difference (MD) with 95% limits of agreement (LOA) (mean difference ± 1.96× standard deviation). This data was then used to construct two Bland-Altman plots, one being mean lactate vs difference in lactate and the other being mean pH vs difference in pH.

Bland and Altman described that when two techniques are compared, with the possibility of one substituting the other, the MD with LOA, rather than just the correlation coefficient between the measurements, will provide a better measure of their agreement, hence why this method was used [3]. All statistical analyses and construction of both graphs were executed using SPSS software v21.

## Results

A total of 220 sepsis patients were identified within the study period. 170 of these children had both an ABG and VBG, with no fluid bolus given between the samples. 110 of these children had to be excluded because the ABG and VBG were taken more than 1 h apart of each other (Fig. 1).

Sixty patients were included overall with a mean age of 4.4 (SD = ±4.4) years. Admission diagnoses are recorded in Table 1. Median VBL was 1.9 mmol/L (interquartile range 1.4–4.7 mmol/L) and median ABL was 1.6 mmol/L (IQR 1.1–3.2 mmol/L). Median venous pH was 7.25 (IQR 7.20–7.32) and median arterial pH was 7.32 (IQR 7.24–7.37). Nine patients (15%) died during admission, with their data still included in this study.

**Fig. 2:**
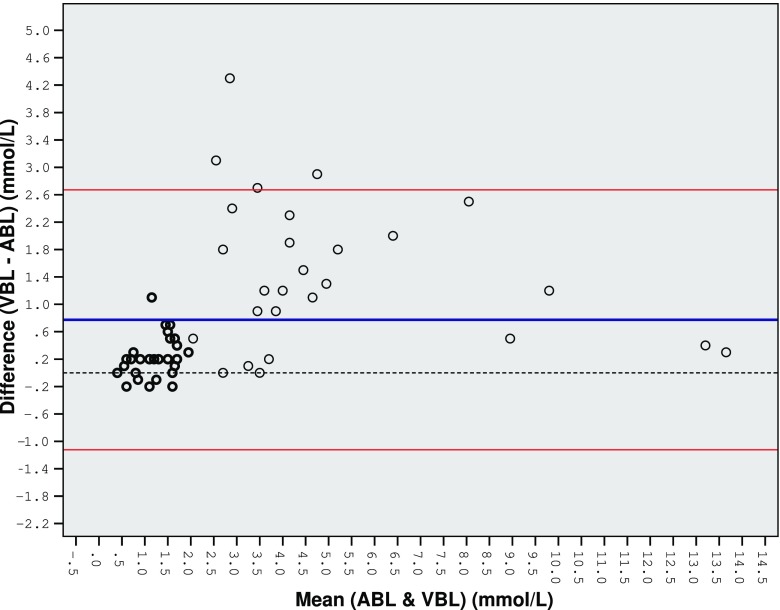
Bland-Altman plot of agreement between venous blood lactate and arterial blood lactate

Figure 2 is a Bland-Altman plot of agreement between venous blood lactate and arterial blood lactate.

**Table 1:** Ages, admission diagnoses and lactate and pH values for all patients, including those that died during admission

Mean age (±SD)	4.4 years (±4.4)
Diagnosis on admission, no. (%)	Sepsis—*n* = 35 (58%) Neutropenic sepsis—*n* = 10 (17%) Septic shock—*n* = 15 (25%)
Median VBL (interquartile range)	1.9 mmol/L (1.4–4.7 mmol/L)
Median ABL (interquartile range)	1.6 mmol/L (1.1–3.2 mmol/L)
Mean difference (±LOA)	0.775 mmol/L (±1.90 mmol/L)
Median venous pH (interquartile range)	7.25 (7.20–7.32)
Median arterial pH (interquartile range)	7.32 (7.24–7.37)
Mean difference (±LOA)	−0.056 (±0.121)
Patients that died during admission, no (%)	9 (15%)

The results circled in black represent patients with an initial venous lactate of 2 mmol/L or less.

Until lactate reaches 2.0 mmol/L, the differences between the two measurements are minor and the agreement is close. As the lactate level increases above 2 mmol/L, this agreement becomes increasingly poor and the differences widen. The LOA are too large clinically (±1.90 mmol/L) to allow venous and arterial blood lactate to be used interchangeably.

Interestingly, the MD and the LOA between the two methods would be a lot smaller if they were derived using only mean lactate values of 2.0 mmol/L or lower (results circled in black), suggesting that venous lactate could be used independently as a measure of sepsis providing the initial result is 2.0 mmol/L or less.

Table 2 is a cross tabulation of categorised venous blood lactate against arterial blood lactate.

**Table 2:** Cross tabulation of categorised venous blood lactate against arterial blood lactate

	Arterial lactate (mmol/L)			
		>2	≤2	Total
Venous lactate (mmol/L)	>2	22	6	28
	≤2	0	32	32
	Total	22	38	60

Venous and arterial blood lactate measurements were categorised as ≤2 mmol/L (indicative of normal) or >2 mmol/L (indicative of abnormal). Categorised venous blood lactate was crosstabulated against arterial blood lactate. All of the patients with arterial blood gases that found lactate to be abnormal (>2 mmol/L), were identified initially by a venous blood lactate of more than 2 mmol/L. None of them had an initial normal venous lactate (≤2 mmol/L).

Of all the patients with a normal arterial blood lactate (≤2 mmol/L), 32 (84.2%) of them had been identified as having an initial normal venous lactate.

**Fig. 3:**
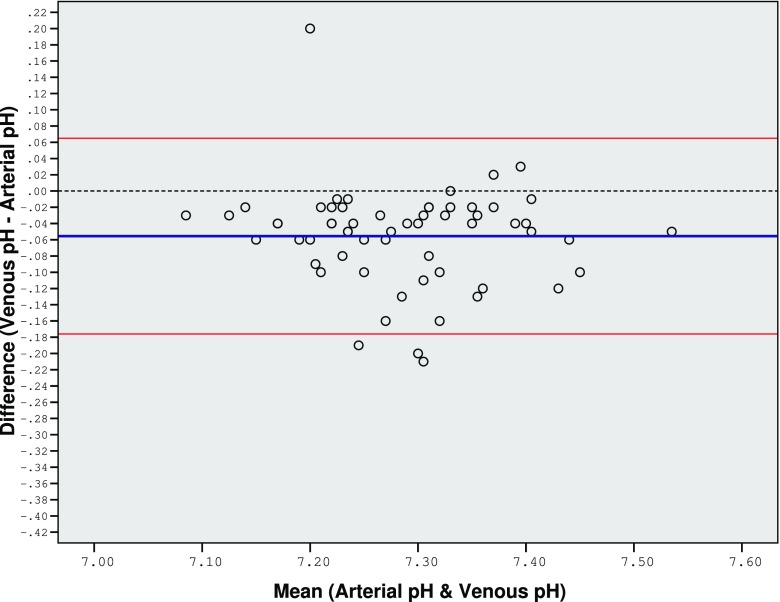
Bland-Altman plot of agreement between venous blood pH and arterial blood pH

Figure 3 is a Bland-Altman plot of agreement between venous blood pH and arterial blood pH.

The MD in pH between both methods was only −0.056 and the majority of the difference values lie within very narrow LOA (±0.121). This close agreement supports the idea that VBGs and ABGs can be used interchangeably when measuring pH.

## Discussion

The PICU in which the study took place was in a large London hospital, serving a very ethnically diverse cohort. The data presented here is likely to be generalisable to other similar PICU departments.

These results demonstrate close agreement between venous and arterial lactate up to the value of 2 mmol/L, where lactate becomes abnormal. After this value the agreement becomes increasingly poor. Similar findings were reported by Bloom et al. [4] who looked at the agreement between arterial and venous lactate in adults, when the initial venous lactate was over 2 mmol/L and also over 4 mmol/L. Middleton et al. [10] found a close agreement with narrow LOA between arterial and venous lactate in adults, with the majority of their patients having an initial venous lactate under 2 mmol/L. Therefore, the use of venous lactate alone to identify and measure sepsis can be advocated, providing the initial measurement is equal to or below 2.0 mmol/L. If the initial measurement is over 2.0 mmol/L an arterial sample must be drawn to confirm a measurement which is potentially over-exaggerated.

There is close agreement between arterial and venous pH, regardless of whether the patient was in acidosis or alkalosis. This finding is consistent with the findings of other studies [2, 10]. However, clinically, due to the wide variation in pH readings during sepsis, pH alone is an inadequate marker of sepsis.

It is important to reflect that haemodynamic compromise associated with sepsis would logically lead to a greater variation in venous and arterial lactate in the presence of severe sepsis, particularly septic shock. This acknowledged hypothesis correlates with the widening difference between venous and arterial lactate at higher values (>2 mmol/L), and must be a consequence of reduced peripheral perfusion in the presence of worsening sepsis. Moreover, this would also be a unique feature of sepsis in comparison with other clinical conditions related to lactic acidosis such as metabolic disorders.

This study has some limitations. The strict inclusion criteria for patient selection significantly reduced the number of patients that were used. It is advised that another study using several centres with PICUs is undertaken to strengthen the conclusions drawn from this study. Apart from age and admission diagnosis, no other patient demographics such as gender, ethnicity and past medical history, including congenital abnormalities, were recorded. Some of these could be potential confounding factors with regard to lactate measurement. The time between the venous and arterial sample being taken varied from 15 minutes to 1 hour. Although no intervention had been given between the samples, it could be suggested that a 1 hour interval may be too long to make reliable conclusions. Ideally each sample would have been taken simultaneously, however, this is neither clinically possible nor ethical. Interestingly, an adult study [4] found that there was still a ‘similar trend but with slightly closer agreement’ between samples taken 15 minutes apart compared to those that were taken 1 hour apart. Finally the duration of which a tourniquet was applied during each venous sample was not measured, something that is thought to affect the initial lactate. However this was not shown to be significant in a trial [7] comparing lactate values between a group in which a tourniquet was not used and a group in which a tourniquet was applied 5 minutes before the sample was taken.

The main strength of this study arises from the fact that this is the first study which assesses the agreement between arterial and peripheral venous lactate in children with sepsis, including a sample of more than 50 patients, making our conclusions significant and reliable. Our study also fortunately included similar numbers of children with normal and abnormal lactate, reducing this bias observed in other studies mentioned previously. The potential to be able to initiate treatment based on a venous lactate measurement alone, providing it is 2 mmol/L or lower, poses great clinical significance in a busy PICU, as valuable time could be saved with minimal distress to the critically ill child. Further studies adhering to our strict inclusion criteria should be undertaken, with larger samples of patients drawn from more PICUs, to reliably confirm and strengthen these conclusions.

## Conclusion

A venous blood gas with a lactate of 2 mmol/L or lower can be used as a surrogate measurement for arterial lactate during early management of sepsis in children. However if this value is above 2 mmol/L, an arterial sample must be taken to confirm the venous result.

## Abbreviations

ABG: Arterial blood gas
ABL: Arterial blood lactate
LOA: Limits of agreement
MD: Mean difference
VBG: Venous blood gas
VBL: Venous blood lactate
